# Editorial: New Insights Into the Biodegradation of Organic Contaminants in Subsurface Ecosystems: Approaches and Achievements of the Multiomics Era

**DOI:** 10.3389/fmicb.2021.650615

**Published:** 2021-05-04

**Authors:** András Táncsics, Mengyan Li, Łukasz Chrzanowski

**Affiliations:** ^1^Department of Molecular Ecology, Hungarian University of Agriculture and Life Sciences, Gödöllö, Hungary; ^2^Department of Chemistry and Environmental Science, New Jersey Institute of Technology, Newark, NJ, United States; ^3^Faculty of Chemical Technology, Poznan University of Technology, Poznań, Poland

**Keywords:** contaminant biodegradation, multi-omics approaches, petroleum hydrocarbons, chlorinated organic compounds, subsurface microbiology

Contamination of the subsurface ecosystems with organic compounds originating from the petrochemical, pharmaceutical and agricultural industries is a persistent and growing problem globally. Due to the world's population growth, more and more complex organic molecules are released to the environment, threatening pristine drinking water reservoirs or soil health.

Despite the enormous amount of data on organic contaminant degrading microbial communities and strains provided by genomic and metagenomic studies, there are still several questions that remain unanswered due to the limitations of using these techniques alone. The combination of “*omics”* technologies gives new perspectives in studying contaminant biodegradation. It makes possible the real “*in-depth analysis*” of contaminant degrading microbial communities by strain-level profiling (Franzosa et al., 2015). Consequently, researchers can reveal the exact role of the uncultivated majority in contaminated environments (Solden et al., 2016). This uncultivated majority is often referred as the “microbial dark matter” (Rinke et al., 2013), which terminology will probably be outdated soon, due to the extensive use of “*omics*” technologies in environmental microbiological studies. Nevertheless, there are several other questions of contaminant biodegradation, which should be addressed. The “*multi-omics*” approaches can help us to better understand how microbes can cope with high or low concentrations of organic contaminants. Other questions are how the allochthonous microbes affect the indigenous microbial community during a bioremediation process or how changes in the environmental parameters (e.g., temperature or electron acceptor availability) affect microbial community composition or activity in a contaminated environment. The aim of the present Research Topic was to widen our knowledge regarding these issues.

Some organic contaminants occur in low concentrations in the environment. An interesting question is how degrading microbes adapt to these low substrate concentrations. Do they focus on a single substrate or they de-repress regulation of other catabolic pathways? To address this question, Marozava et al. conducted column experiments with the benzoate-degrading *Geobacter metallireducens*. By using the combination of metagenomics and metaproteomics it was shown that below 0.2 mM benzoate concentration the complete upregulation of the toluene-degrading pathway was observable, although toluene was not present in the columns. The authors suggested an updated model for the regulation of catabolic pathways of *G. metallireducens* as a response to substrate concentration. Accordingly, as a survival strategy *G. metallireducens* seems to scavenge the environment for alternative substrates under carbon-limiting conditions.

Chlorinated organic compounds are still frequent groundwater contaminants worldwide. Since only members of the class *Dehalococcoidia* are known thus far to catalyze complete dehalogenation of PCE to ethene, these bacteria are still in focus of research. Franke et al. investigated *Dehalococcoides mccartyi* strain BTFo8, which is able to perform complete reductive dehalogenation of PCE to ethene, and it is capable of dehalogenating vicinal halogenated alkanes as well. The genome of *D. mccartyi* strain BTFo8 contains 20 reductive dehalogenase genes. Although the putative functional annotation of these genes was performed, their exact biochemical activity remained unclear. To address this issue, the authors characterized the reaction mechanisms during dehalogenation of PCE, cDCE, VC, and 1,2-dichloroethane using dual element (C/Cl) compound specific stable isotope analysis combined with proteomic analysis. The exact role of the different dehalogenases in the processes was also revealed.

Besides chlorinated compounds, aromatic hydrocarbons are another group of major aquifer contaminants. The main rate-limiting factor of their biodegradation in subsurface environments is the availability of electron acceptors. It is also well known that different electron acceptors support the activity of distinct degraders. Still there is a lack of knowledge on how nitrite affects degrading communities. Zhu et al. compared toluene-degrading microbial communities with different nitrate and nitrite availabilities. For that, DNA-stable isotope probing was coupled with 16S rRNA gene amplicon sequencing. It was shown that nitrite not just modulated the microbial communities responsible for toluene degradation but also influenced how nitrate reduction proceeded.

Bioaugmentation of petroleum hydrocarbon contaminated environments, especially soils, is still a frequent bioremediation process. Inoculants containing members of the genus *Rhodococcus* are top bioaugmentation agents, since these bacteria are masters of catabolic versatility, and most importantly, they are excellent alkane-degraders (Larkin et al., 2005). Pacwa-Płociniczak et al. investigated the effect of two *R. erythropolis* strains on petroleum hydrocarbon removal and bacterial community composition in soil. Based on the results of 16S rRNA gene amplicon sequencing and metagenome functional content prediction, the authors concluded that the *Rhodococcus* strains were present in the inoculated soils only at the beginning of the experiment, thus hydrocarbon loss in the analyzed soil resulted from the activity of autochthonous microorganisms. Nevertheless, the inoculated *Rhodococcus* strains induced changes in the indigenous microbial community, positively affecting degradation efficiency.

High molecular weight polycyclic aromatic hydrocarbons (HWM PAHs) are among the most harmful organic compounds threatening human health and the environment. Miao et al. investigated the biodegradation of indeno[1,2,3-cd]pyrene (IcdP) which contains six aromatic rings. It was shown that a *Rhodococcus aetherivorans* strain was a highly efficient IcdP degrader. The authors analyzed the genome of *R. aetherivorans* strain IcdP1 and investigated the expression of genes most probably playing key role in the initiation of the degradation process. As a result the authors revealed that this strain used multiple aromatic ring-hydroxylating dioxygenases for ring activation and the initial hydroxylation occurred at least at the 1,2 and 7,8 positions of the molecule.

Bin Hudari et al. investigated how aquifer thermal energy storages (ATES) may affect subsurface microbial communities. For this, a laboratory microcosm study was performed with a hydrocarbon-degrading microbial community from a sulfidic aquifer. Microcosms were spiked with ^13^C-labeled acetate and incubated at temperatures between 12 and 80°C. Subsequently, microbial community analyses were performed by 16S rRNA gene amplicon sequencing. Due to an initial oxygen exposure, a shift from oxic to sulfidogenic conditions was observable. Distinct microbial communities developed under oxic and anoxic conditions as well as at different temperatures. Up to 38°C both aerobic and anaerobic acetate degradation was observable, while mineralization occurred mainly aerobically between 45 and 60°C. Above 45°C the activity of thermophilic sulfate reducers was not detected. The authors concluded that temperature may affect dissolved organic carbon mineralization rates in ATES.

The large and diverse candidate phylum *Saccharibacteria* is often referred as part of the “microbial dark matter” (Marcy et al., 2007; Murugkar et al., 2020). Earlier a group of them was found to be abundant in diesel fuel contaminated soil hinting at their role in petroleum hydrocarbon degradation. Figueroa-Gonzalez et al. investigated petroleum hydrocarbon-degrading enrichment cultures, in which *Saccharibacteria* were abundant community members. Genome resolved metagenomics enabled to reconstruct two almost identical *Saccharibacteria* genomes. The acquired genome data revealed small genome size (~1 Mbps) without the capability of hydrocarbon degradation. It was concluded that these organisms can be highly abundant scavengers acting as organic carbon sinks in petroleum hydrocarbon-fueled environments.

Laczi et al. aimed to review the anaerobic hydrocarbon degradation processes, the most important hydrocarbon degraders, and their diverse metabolic pathways. The review article primarily focuses on the latest findings achieved by high-throughput multi-omics techniques.

## Author Contributions

All authors listed have made a substantial, direct and intellectual contribution to the work, and approved it for publication.

## Conflict of Interest

The authors declare that the research was conducted in the absence of any commercial or financial relationships that could be construed as a potential conflict of interest.
